# Amine modification of calcium phosphate by low-pressure plasma for bone regeneration

**DOI:** 10.1038/s41598-021-97460-8

**Published:** 2021-09-09

**Authors:** Joe Kodama, Anjar Anggraini Harumningtyas, Tomoko Ito, Miroslav Michlíček, Satoshi Sugimoto, Hidekazu Kita, Ryota Chijimatsu, Yuichiro Ukon, Junichi Kushioka, Rintaro Okada, Takashi Kamatani, Kunihiko Hashimoto, Daisuke Tateiwa, Hiroyuki Tsukazaki, Shinichi Nakagawa, Shota Takenaka, Takahiro Makino, Yusuke Sakai, David Nečas, Lenka Zajíčková, Satoshi Hamaguchi, Takashi Kaito

**Affiliations:** 1 Department of Orthopaedic Surgery, Osaka University Graduate School of Medicine, 2-2 Yamadaoka, Suita, Osaka 565-0871 Japan; 2 Center for Atomic and Molecular Technologies (CAMT), Graduate School of Engineering, Osaka University, 2-1 Yamadaoka, Suita, Osaka 565-0871 Japan; 3 Center for Accelerator Science and Technology, National Nuclear Energy Agency of Indonesia (BATAN), Jalan Babarsari Kotak Pos 6101 ykbb, Yogyakarta, 55281 Indonesia; 4 Department of Physical Electronics, Faculty of Science, Masaryk University, Kotlarska 2, 61137 Brno, Czech Republic; 5 Bone and Cartilage Regenerative Medicine, Graduate School of Medicine, The University of Tokyo, 7-3-1 Hongo, Bunkyo-ku, Tokyo, 113-8655 Japan; 6 CEITEC – Central European Institute of Technology, Brno University of Technology, Purkynova 123, Brno, 61200 Czech Republic; 7 Department of Condensed Matter Physics, Faculty of Science, Masaryk University, Kotlarska 2, Brno, 61137 Czech Republic

**Keywords:** Translational research, Biomedical materials, Implants

## Abstract

Regeneration of large bone defects caused by trauma or tumor resection remains one of the biggest challenges in orthopedic surgery. Because of the limited availability of autograft material, the use of artificial bone is prevalent; however, the primary role of currently available artificial bone is restricted to acting as a bone graft extender owing to the lack of osteogenic ability. To explore whether surface modification might enhance artificial bone functionality, in this study we applied low-pressure plasma technology as next-generation surface treatment and processing strategy to chemically (amine) modify the surface of beta-tricalcium phosphate (β-TCP) artificial bone using a CH_4_/N_2_/He gas mixture. Plasma-treated β-TCP exhibited significantly enhanced hydrophilicity, facilitating the deep infiltration of cells into interconnected porous β-TCP. Additionally, cell adhesion and osteogenic differentiation on the plasma-treated artificial bone surfaces were also enhanced. Furthermore, in a rat calvarial defect model, the plasma treatment afforded high bone regeneration capacity. Together, these results suggest that amine modification of artificial bone by plasma technology can provide a high osteogenic ability and represents a promising strategy for resolving current clinical limitations regarding the use of artificial bone.

## Introduction

Autogenous iliac bone grafting remains the gold standard for repairing large bone defects caused by trauma or tumors, or in spinal fusion surgeries. However, the amount of autograft that can be harvested is limited and the harvesting procedure can cause donor site morbidity^1^. To overcome these limitations, the use of artificial bone, in combination with autografts is prevalent. However, the widespread use of artificial bone is hampered by its lack of satisfactory osteogenic ability, despite its superiority in terms of bone conduction and availability^2^.

To enhance functionality, surface modification on biomaterials mediated by plasma technology has gained considerable attention. Functional groups created by plasma polymerization (i.e., polymer formation via plasma discharges) can provide selected surface properties such as hydrophilicity/hydrophobicity, cytocompatibility, and bacterial resistance to meet different clinical needs^3^. It was previously reported that plasma treatment of interconnected porous hydroxyapatite (HA) artificial bone with O_2_/He gas could improve surface hydrophilicity and promote the osteogenic differentiation of rat bone marrow stromal cells (BMSCs)^4–6^. However, the O_2_/He plasma-treated HA exerted only minimal effects in vivo, possibly owing to the instability of the generated hydroxyl (–OH) groups. In the present study, we focused on the addition of amino groups or amines (–NH_*x*_ with *x* = 0–2), which has been suggested to promote cell attachment^7,8^. Unlike in the earlier studies^5,6^, we used β-TCP, rather than HA. Both are calcium phosphates widely used for artificial bone, but β-TCP is known to have a comparative advantage over HA as being absorbed in vivo (biodegradable)^9^. Using a gas mixture of CH_4_, N_2_, and He for plasma treatment, we successfully generated amine-containing carbon polymer on the surfaces of β-TCP, including the surfaces of interconnected inner pores of porous β-TCP artificial bones. Here, we demonstrate the effects of amine modification on β-TCP by low-pressure plasma for in vitro cell adhesion, osteogenic differentiation, and in vivo bone regeneration with a rat calvarial defect model.

## Materials and methods

### Materials and reagents

Dense and porous disks of β-TCP [Ca_3_(PO_4_)_2_] were provided by Coors Tek KK (Tokyo, Japan). All the dense and porous disks used in this study had the same dimensions of 5 mm in diameter and 2 mm in height (φ 5 mm × h 2 mm). The porous disks had well-organized interconnected structures with a porosity of 72–78%, an average pore diameter of 150 μm, and an average diameter of interconnected passages of 40 μm^10^.

The following culture media were used for in vitro experiments: (1) growth medium (GM) comprising α Eagle’s minimal essential medium (α-MEM, Gibco, ThermoFisher Scientific, Waltham, MA, USA) supplemented with 10% fetal bovine serum (Sigma-Aldrich, St. Louis, MO, USA) and 1% antibiotic–antimycotic solution (Sigma-Aldrich); (2) osteogenic differentiation medium (ODM) consisting of GM supplemented with 50 μg/ml l-ascorbic acid 2-phosphate (Sigma-Aldrich), 10 mM β-glycerol phosphate (Merck KGaA, Frankfurt, Germany), and 10 nM dexamethasone (Sigma-Aldrich).

Cell Counting Kit-8 (CCK-8, Dojindo Molecular Technologies, Kumamoto, Japan) was used for the cell proliferation assay. LabAssay ALP (FUJIFILM Wako Pure Chemical Corp., Osaka, Japan) was used for the evaluation of alkaline phosphatase (ALP) activity and BCIP/NBT Color Development Substrate (Promega Corp., Madison, WI, USA) was used for ALP staining. M-PER and Pierce Rapid Gold BCA Protein Assay Kits (Thermo Fisher Scientific) were used for total protein extraction and quantification, respectively. K-CX AT solution (Falma Co., Tokyo, Japan) was used for decalcification of in vivo specimens.

### Plasma polymerization of β-TCP disks

**Figure 1:**
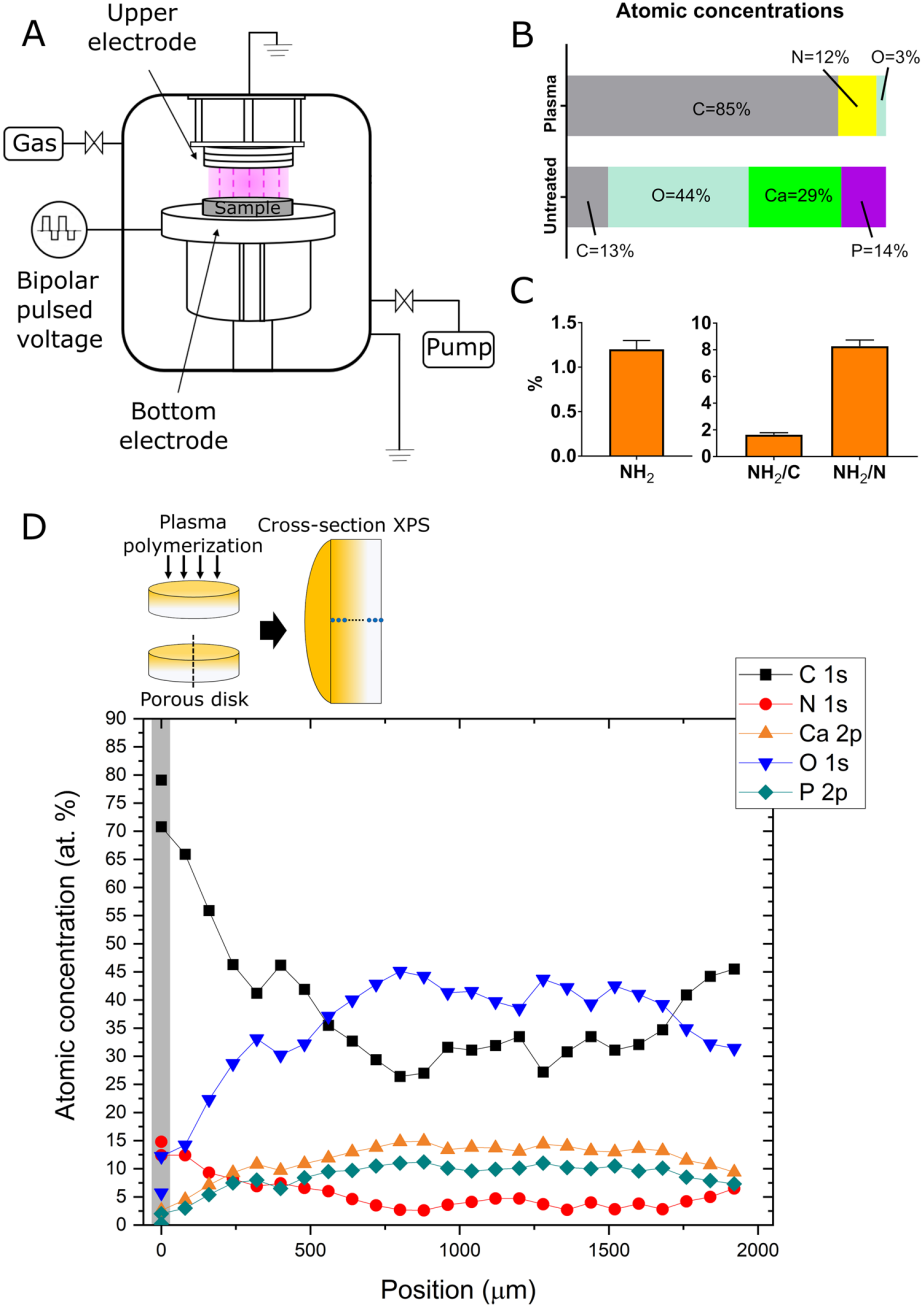
Plasma polymerization system and characterization of plasma-polymerized films. (**A**) Schematic diagram of the pulsed-plasma deposition system used in this study. Samples were placed on the bottom metal electrode, which was powered by high-voltage bipolar pulse voltages with a peak-to-peak voltage of 2.2 kV. The power, repetition frequency, and duty cycle were 15 W, 5 kHz, and 1%, respectively. The plasma was generated from a gas mixture of CH_4_, N_2_, and He with flow rates of 10, 20, and 10 sccm, respectively, and a gas pressure of 70 Pa. (**B**) The atomic concentration ratios on a plasma-treated dense β-TCP disk surface (top) and those on an untreated dense β-TCP disk surface (bottom). More precise values are given in the main text. (**C**) The relative number of primary amines (–NH_2_) among all atoms excluding hydrogen (left) and the number ratios of primary amines to C or N atoms (right) on the surface of the plasma-polymerized film deposited on a dense β-TCP disk. In (**B**,**C**), all measurements were triplicate. (**D**) The profiles of relative atomic concentrations along the center axis of a plasma-treated porous β-TCP disk, indicating plasma polymerization of the inner pore surfaces due to the penetration of plasma-generated deposition precursors through the interconnected pores from the plasma-treated disk surface. After a single side of a porous β-TCP disk was plasma-treated, it was cut in half through its center and the relative atomic concentrations were measured at 25 points along the center axis. The horizontal axis of the figure represents the position along the center axis measured from the plasma-exposed top surface of the disk. In each case above, the plasma treatment time was 30 min. Schematic diagrams (**A** and the top part of **D**) were drawn using Microsoft PowerPoint 2016.

Plasma polymerization was performed with a bipolar pulsed-plasma deposition system, as shown in Fig. 1A. The details of the system are described elsewhere^11–14^. The β-TCP disks were placed on the bottom metal (molybdenum) electrode, 190 mm in diameter, connected to the bipolar high-voltage power supply. The applied bipolar pulse voltages were 1.1 and − 1.1 kV (peak-to-peak voltage of 2.2 kV) and the pulse duration was 1 μs for each positive or negative pulse. The power, repetition frequency, and duty cycle were 15 W, 5 kHz, and 1%, respectively. The time lapse between a positive pulse and the subsequent negative pulse was 100 μs. The upper metal (aluminum) electrode, 80 mm in diameter, was grounded and the distance between the two electrodes was 38 mm. For plasma polymer deposition, the discharge was generated in a CH_4_/N_2_/He gas mixture with flow rates of 10, 20, and 10 sccm, respectively, and a gas pressure of 70 Pa. For biological (i.e., in vitro and in vivo) experiments, dense disks were treated only on one side for 30 min whereas porous disks were treated on both sides for 60 min (i.e., 30 min each). This is because dense disks were used only for in vitro experiments in this study, where cells were places only on one side of the disk, whereas porous disks were used only for in vivo experiments in this study, where all sides of the disk were exposed to the animal tissues. In either one-side or both-side plasma treatment, the sidewall of a disk was exposed to the plasma and therefore plasma treated. For non-biological experiments (e.g., physical or chemical characterization of plasma-polymerized films), the disk was plasma treated only on one side for 30 min and the film deposited on the plasma-facing surface or the inner pore surfaces was examined.

### Plasma polymer film characterization

The chemical compositions of untreated or plasma-treated β-TCP surfaces were analyzed by X-ray photoelectron spectroscopy (XPS) using ESCA-850 (Shimadzu Co., Kyoto, Japan) with a non-monochromatized Mg-K_α_ (1253.6 eV) X-ray source at Osaka University, for which the pass energy was 75 eV, the photoelectron take-off angle was 90°, and the spot diameter was 8 mm (90% uniformity), or by high-resolution XPS using PHI Quantera II (ULVAC-PHI, Inc., Chigasaki, Japan) with a monochromatic Al-Kα (1486.6 eV) X-ray source at the Foundation for Promotion of Material Science and Technology of Japan (MST), for which the pass energy was 112 eV, the photoelectron take-off angle was 45°, and the spot diameter was 100 μm.

The thickness of a deposited polymer film was evaluated by the standard ellipsometry^15,16^, using data acquired with the V-VASE Ellipsometer (J.A. Woolam, Lincoln, NE, USA) at Central European Institute of Technology (CEITEC), Brno University of Technology, in the spectral range from 0.75 to 6.5 eV at four angles of incidence 60°, 65°, 70° and 75°.

The detection of primary amine groups (–NH_2_) on plasma-treated disk surfaces was performed using the standard derivatization with 4-trifluoromethyl-benzaldehyde (TFBA), according to the published method^17,18^. The derivatization reactions of TFBA vapors with plasma-treated disk surfaces were allowed to occur in an Ar atmosphere (Ar flow rate of 60 sccm at atmospheric pressure) at room temperature (approximately 25 °C) for 4 h inside the glove box. The relative concentrations of primary amines on the sample surfaces were determined by the detection of fluorine (F) atoms of TFBA with ESCA-850 XPS analysis after the derivatization reactions.

The surface morphologies of untreated and plasma-treated porous β-TCP disks were observed with a scanning electron microscope (SEM) (S-4800, Hitachi, Ltd., Tokyo, Japan) at Osaka University.

### Cells

Rat BMSCs were obtained from the bone shafts of the femora of four 3-week-old green fluorescent protein (GFP)-transgenic male Sprague–Dawley rats (SD-Tg (CAG-EGFP) rats, Japan SLC, Hamamatsu, Japan). Following the sacrifice of the rats using CO_2_ inhalation, both ends of the femur were removed from the epiphysis; the marrow was flushed out using 10 ml of GM expelled from a syringe through a 21-gauge needle according to the previously described method^19^. The released cells were collected in two 100 mm culture dishes containing 15 ml of GM. The medium was changed after 24 h to remove hematopoietic cells and renewed twice weekly. Cultures were maintained in a humidified atmosphere of 95% air with 5% CO_2_ at 37 °C. When the cells reached 80–90% confluency they were washed with phosphate-buffered saline (PBS) and trypsinized with 1% trypsin-ethylenediaminetetraacetic acid (EDTA). Following centrifugation for 5 min at 400*g*, the cells were resuspended and plated at a density of 3.6 × 10^4^/cm^2^. After again reaching confluency, cells were collected and stored at − 80 °C (Passage 1). Prior to in vitro experiments, stocked cells were thawed and resuspended in 15 ml of GM, then plated in a 100 mm dish and cultured for three days to reach 80–90% confluency (Passage 2).

### Cell adhesion assay

A centrifugation cell adhesion assay was performed according to a published method^20^. Cell suspension (5 × 10^3^ cells/35 μl GM) was gently dropped on each dense β-TCP disk surface to form a centroclinal water drop and incubated for 30 min in a 24 well culture plate to initiate adhesion. Then, 1 ml of PBS was gently added to each well containing a cell-adhered disk and macro fluorescence photos were taken with the Leica AF6000 Fluorescence Imaging System (Leica Microsystems, Wetzlar, Germany. Exposure: 1000 ms; Gain: 2.7; Binning 2 × 2; Magnification: 12.6×) to quantify the initial adherent cells. After this, the cell adhered disks were embedded into a 48-well culture plate containing 100 μl of Vaseline (KENEI Pharmaceutical Co., Ltd., Osaka, Japan) in each well. After filling each well with PBS, the plate was sealed with a sealing tape and set upside-down on a centrifuge (PlateSpinII, Kubota Corp., Tokyo, Japan) and centrifuged at 10*g* for 5 min to detach weakly adherent cells. After the centrifugation, the detached cells were slowly aspirated and each well was carefully filled with 200 μl of PBS. Macro fluorescence photos of the plate were taken again under the same conditions as those prior to the centrifugation. Automatic cell counting was performed with the macro fluorescence photos and ImageJ software^21^. The adhesion rate is defined as the ratio of the number of cells attached to the surface after centrifugation to that before centrifugation.

### Morphology analysis of adhered cells

Cell suspension (5 × 10^3^ cells/35 μl GM) was gently dropped on dense β-TCP disks and incubated for 3 h in a 24-well culture plate to initiate adhesion. Then, the wells were slowly filled with 1 ml of GM, and the incubation was continued for another 24 h. Macro fluorescence photos of random areas of the culture wells containing incubated cells were then obtained. The open-source software CellProfiler (www.cellprofiler.org)^22^ was used for sorting cells and cell morphology analysis. In particular, the cell area was measured to quantify the spreading of attached BMSCs. Two cell-shape descriptors (circularity and solidity) were investigated; the circularity indicates the closeness of the cell shape to a perfect circle, and the solidity is an index to quantify the amount and size of concavities of the cell^23^:

1 $$ Circularity = \frac{4\pi A}{{P^{2} }} $$

where *A* is the cell area and *P* is the perimeter.

2 $$ Solidity = \frac{A}{ConvexA} $$

where *ConvexA* is the area of the smallest convex hull that contains the cell.

### Cell proliferation assay

To balance the initial cell count of adherent cells on untreated and plasma-treated β-TCP disks, two different concentrations (5 × 10^3^ cells/35 μl GM for the untreated group and 3 × 10^3^ cells/35 μl GM for the plasma-treated group) of cell suspension were dropped on dense untreated and plasma-treated β-TCP disks in a 48-well plate. After incubating for 30 min, 500 μl of GM was slowly added to each well. At day 1, 3, 5, 7, 11, and 14, 50 μl of CCK-8 solution was added to each well and the plate was incubated for 2 h, then 100 μl of GM from each well was transferred into a 96-well plate. The optical density at 450 nm was measured using a spectrophotometer (Multiskan GO, Thermo Fisher Scientific).

### Osteogenic differentiation assay

Cell suspension (2 × 10^4^ cells/35 μl GM ) was dropped on dense β-TCP disks and incubated for 30 min in a 48-well plate to initiate adhesion. Then, 500 μl of GM was slowly added into each well, incubated for 24 h, and the culture medium was replaced with ODM for osteogenic differentiation. The subculture was maintained for another four days. After washing the disks twice with PBS, the attached cells were fixed using 500 μl of 4% paraformaldehyde (PFA) for ALP staining, according to the manufacturer’s instructions (Promega Corp.). For ALP activity, 60 μl of M-PER was added to each well and the cells were detached from the disks using a mini scraper. After lysing the cells for 5 min, the supernatant was collected for ALP and total protein assays. An ALP activity unit was defined as the release of 1 nmol p-nitrophenol per min of incubation at 37 °C for each β-TCP disk. The total protein content of each sample was measured to standardize the ALP activity values.

### Rat calvarial defect model

A total of 20, 8-week-old male Sprague–Dawley rats (Charles River Laboratories Japan, Yokohama, Japan) were used to generate the calvarial defect model. Anesthesia was maintained by intraperitoneal injection of a mixture of 0.15 mg/kg medetomidine, 2.0 mg/kg midazolam, and 2.5 mg/kg butorphanol after introducing anesthesia by inhalation of 5% isoflurane. A 1.5 cm longitudinal incision was made at the center of the vertex and two full-thickness bone defects with a diameter of 5 mm were then carefully created using a high-speed trephine burr under constant irrigation with saline to avoid heat injury of the surrounding tissue. The plasma-treated and untreated β-TCP disks were implanted in the right and left defects, respectively. The rats were given free access to water and food after the surgery. The rats were sacrificed at postoperative 3 (n = 5) and 6 (n = 15) weeks by CO_2_ inhalation. Microfocus computed tomography (micro-CT) and histological analyses were performed to evaluate new bone formation in the inner pores of porous β-TCP disks at 3 and 6 weeks postoperatively.

### Microfocus computed tomography (micro-CT)

Harvested specimens were fixed in 10% buffered formalin, dehydrated and degreased using a graded ethanol series, and stored in 70% ethanol at 4 °C for micro-CT scanning (Skyscan 1272 micro-CT, Bruker, Kontich, Belgium), which was performed with the following parameters: camera binning = 2 × 2, source voltage = 80 kV, source current = 125 μA, image pixel size = 4 μm, rotation step = 0.6°, and filter = Al 1 mm. Image analysis was performed using CTAN software (Version 1.18.8.0+, Brucker). Micro-CT images of the specimens were compared with the results of histological evaluation of the specimens, discussed below, and the difference in image intensities between the newly formed bone and residual β-TCP was identified. Using this information, three-dimensional (3D) images of newly formed bones inside the porous β-TCP disks were reconstructed from the Micro-CT images of the specimens.

### Histological evaluation

After micro-CT scanning, the specimens were demineralized using K-CX AT solution at 4 °C, cleared in xylene, and embedded in paraffin. Then, several sections of 4 μm thickness were cut off from the center of each specimen and stained with hematoxylin and eosin (H&E).

### Statistical analysis

Statistical analysis was performed using GraphPad Prism version 7.04 for Windows (GraphPad Software, San Diego, CA, USA) applying the Mann–Whitney U test for non-parametric data, Student’s or Welch’s t-test for parametric data, and Wilcoxon matched-pairs signed-rank test for in vivo results. The values are presented as mean ± standard deviation (SD). The differences were considered statistically significant for *p* value < 0.05.

### Ethic declarations

All animal work was approved by The Animal Experimental Committee of Osaka University Graduate School of Medicine (01-070-000) and restrictedly followed ARRIVE guidelines and the National Institutes of Health Guide for the Care and Use of Laboratory Animals^24^.

## Results

### Plasma polymerization on β-TCP disks

A bipolar pulsed-plasma deposition system developed at Osaka University was used to perform plasma polymerization on dense and porous β-TCP disks. The schematic diagram of the system is given in Fig. 1A. The plasma was generated with a CH_4_/N_2_/He gas mixture with flow rates of 10, 20, and 10 sccm, respectively, and a gas pressure of 70 Pa. Methane (CH_4_) was used to form an organic polymer, nitrogen (N_2_) was to form amine groups, and helium (He) was to lower the plasma ignition voltage via Penning ionization. The plasma polymerization conditions used in this study, including the selection of the discharge gases and their mixing ratio, were selected to attain stable plasma discharge, a reasonable film deposition rate, and a relatively high amine concentration in the deposited film, using the gas species and discharge conditions that the authors are accustomed to operating the plasma system with for various other plasma polymerization applications^13,25^. Although the conditions used in this study were locally optimized (i.e., by varying the discharge parameters around those presented in this article), its global optimization with different combinations of organic and nitrogen-compound gaseous species has not been performed yet and is beyond the scope of the present study.

The chemical compositions of the untreated dense β-TCP disk obtained from XPS are Ca: P: O: C = 28.5 ± 0.2: 14.0 ± 1.5: 44.0 ± 0.6: 13.5 ± 2.2 (at.%), and those of the untreated porous β-TCP disk are Ca: P: O: C = 18.9 ± 1.1: 10.8 ± 0.6: 42.3 ± 3.9: 28.0 ± 1.6 (at.%). The measurements were performed in triplicate, i.e., n = 3. No nitrogen (N) was observed on the disk surfaces. It should be noted that hydrogen (H) cannot be detected by XPS and carbon (C) on the surface is essentially a contaminant from ambient air. Compared with the stoichiometric ideal β-TCP (Ca:P:O = 23.1:15.4:61.5), both dense β-TCP disk (Ca:P:O = 33.0:16.1:50.9) and porous β-TCP disk (28.8:16.4:54.9) have Ca-rich compositions. The difference in chemical compositions between dense and porous β-TCP disks is considered to be caused by their different manufacturing processes.

The chemical compositions of the plasma-treated dense β-TCP disk obtained from XPS are Ca: P: O: C:N = 0.0 ± 0.0: 0.0 ± 0.0: 2.9 ± 0.2: 85.2 ± 0.1:11.9 ± 0.1 (at.%, n = 3). The N to C ratio of the deposited film is, therefore, N/C = 14.0%. The fact that neither Ca nor P was observed indicates that the deposited film was sufficiently thick (thicker than several nm at least). The observed small amount of O may indicate oxygen incorporated in the deposited film due to oxygen or water impurities off from the chamber walls of the plasma system during the plasma discharge or water (H_2_O) molecules attached to the polymer surfaces when the sample was exposed to ambient air. The results are summarized in Fig. 1B.

It is not easy to measure the thicknesses of deposited polymer films on β-TCP precisely as the roughness of β-TCP surfaces is far greater than the deposited film thickness. However, we surmise that the deposited polymer thickness on the β-TCP disk under the conditions described above (i.e., 30 min polymerization) was about 40 nm, based on the thickness of the plasma-polymerized film formed on a crystalline (100) Si surface under the same polymerization conditions, which was determined to be 44.2 ± 0.1 nm by ellipsometry.

The relative amount of primary amine (–NH_2_) on a plasma-polymerized film surface was evaluated by the derivatization method^17,18^. Figure 1C shows the relative number of N atoms that form primary amine groups on the surfaces of plasma-polymerized films deposited on dense β-TCP disks 2 days after the film deposition. The left graph shows the atomic percentage of such N atoms among all surface atoms excluding hydrogen ( i.e., the relative number of primary amines among all surface atoms excluding hydrogen), which is 1.2 ± 0.1 (at.%, n = 3). The right graph shows the number ratios of primary amines to the surface C or N atoms, i.e., NH_2_/C = 1.6 ± 0.1 and NH_2_/N = 8.3 ± 0.5 (at.%, n = 3). It is seen here that about 8% of all deposited N atoms formed primary amines in the plasma-polymerized film. The remaining N atoms were most likely to form either secondary amines (-NH) or tertiary amines, i.e., nitrogen atoms bonded only with carbon atoms.

To observe whether the polymer depositing plasma penetrated the interconnected pores of artificial bone and coated their inner surfaces, we performed a cross-section analysis of a plasma-treated porous β-TCP disk. In this analysis, a single-side treated porous β-TCP disk was cut in half at the center and the cross sections were scanned with high-resolution XPS. The measurements were performed on 25 spots with a diameter of 100 μm along the center axis of the cross-section, as presented in Fig. 1D. The vertical and horizontal axes represent the percentages of atomic concentrations and the position along the center axis measured from the plasma-exposed top surface of the disk. We treated only a single side of the disk such that the penetration direction becomes clear. The untreated side of the disk was in direct contact with the electrode surface of the plasma system and not directly exposed to the plasma.

As seen in Fig. 1D, the C and N concentrations are the highest on the plasma-treated surface (i.e., at position 0) and decrease gradually toward the center. The fact that their concentrations slightly higher near the untreated side (i.e., near position 2 mm) suggests that some polymerizing gas entered from this side through a gap between the bottom surface of the disk and the electrode. Because the porosity of the disk is about 75% (i.e., 75% of the disk volume is void), we expect that the area ratio of the inner pore surface to the β-TCP bulk on the cross section is approximately 75^2/3^: 25^2/3^ = 68: 32. We also note that the variation of atomic concentration ratios on the cross section is higher than the SD of the atomic concentration ratios on the untreated β-TCP surface given above. This is because of the random spatial distribution of inner pores, whose average diameter is 150 μm, inside the disk whereas the diameter of each measurement spot is 100 μm.

As discussed earlier, the atomic concentration ratios of untreated β-TCP were about Ca: P: O: C: N = 19: 11: 42: 28: 0 (at.%). Even in a deeper region of the cross section (e.g., the region from 800 to 1700 μm) of Fig. 1D, the atomic concentrations of C and N are higher than those of untreated β-TCP, and those of Ca and P are lower, which indicates that plasma-polymerized films were also deposited on the inner pore surfaces of the deep region of the disk. Plasma-treated porous β-TCP disks used for in vivo experiments of this study were treated on both sides (for 30 min each), as discussed before, so we expect the inner pores of those disks are well coated with plasma-polymerized films.

**Figure 2:**
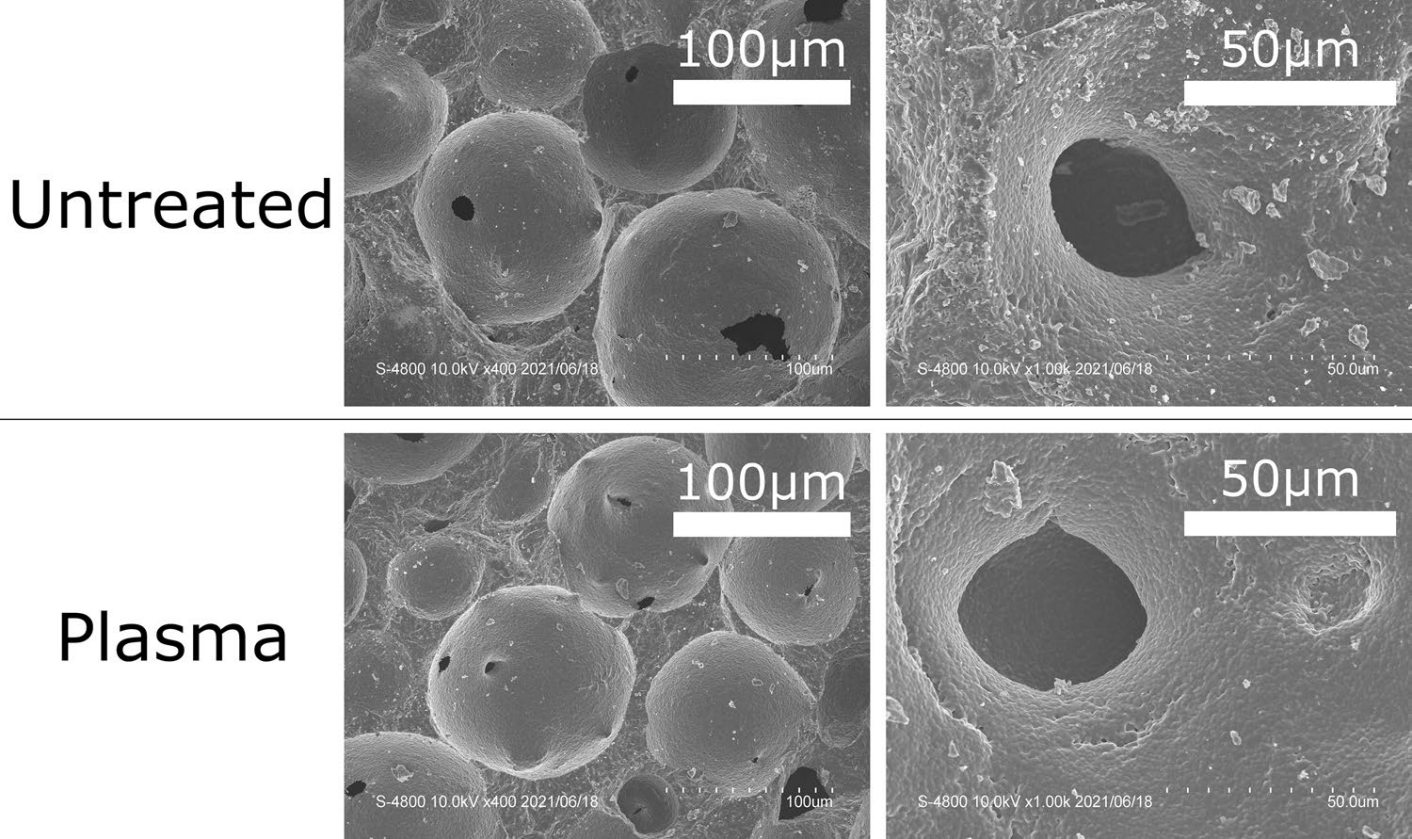
SEM images of the outer surfaces of untreated (top) and plasma-treated (bottom) porous β-TCP disks. Low magnification =  × 400 (left). High magnification =  × 1000 (right).

SEM photos of outer surfaces of untreated and plasma-treated porous β-TCP disks are shown in Fig. 2. Plasma polymerization did not cause structural changes in the porous disks. Typical interconnecting channels are shown in photos at 1000×  magnification. The deposited polymer is not visible here because its thickness (about 40 nm, as discussed above) is far smaller than the scales of these images.

### Effects of plasma treatment on cell behaviors

First, we confirmed that the plasma treatment enhanced the hydrophilicity of β-TCP, which was originally hydrophobic. As a result, cell suspension dropped on plasma-treated porous disks infiltrated quickly, and the cells therein adhered to the walls of their deep inner pores (Supplementary Movie 1, Supplementary Fig. 1).

**Figure 3:**
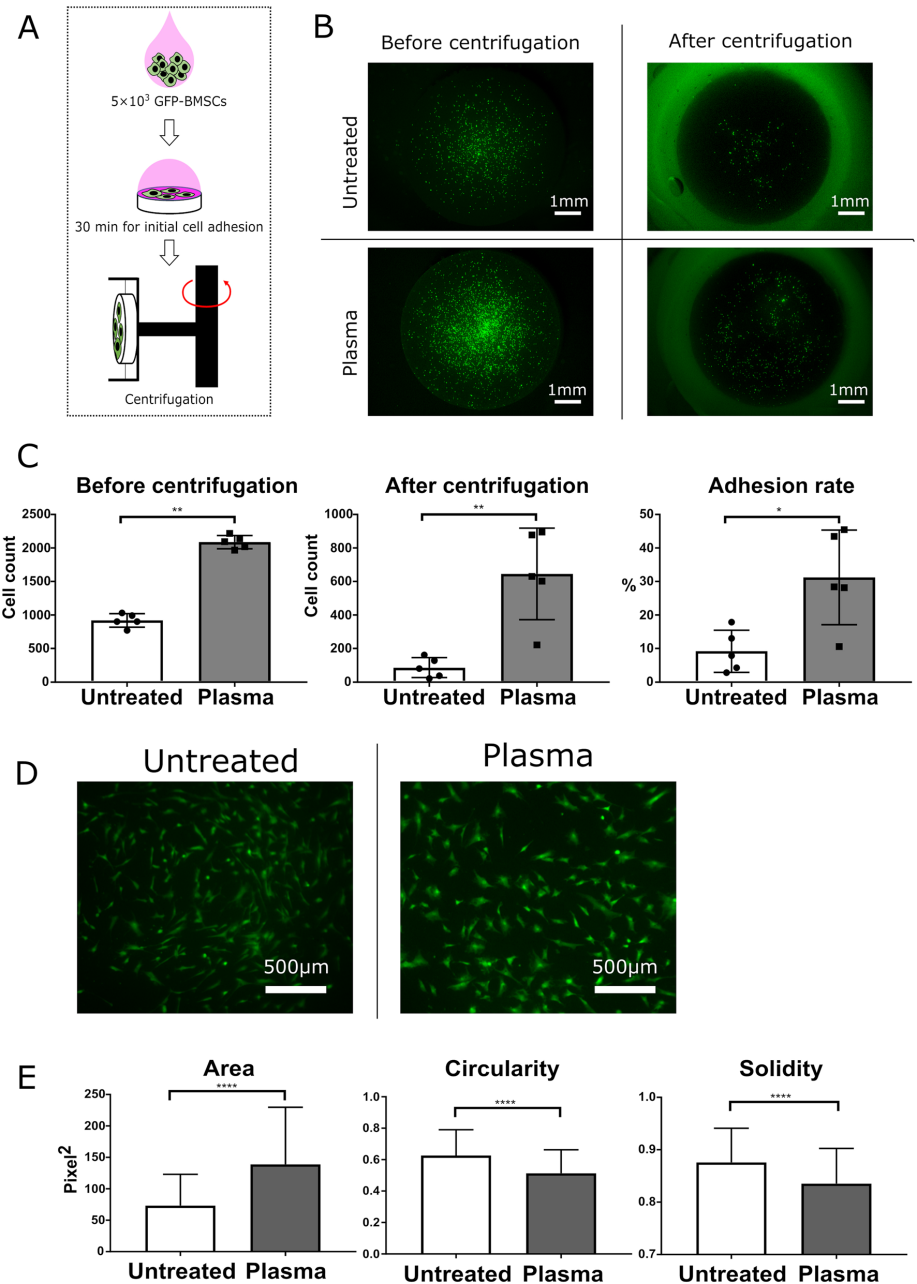
Cell adhesion and morphology assays. (**A**) Schematic diagram (drawn by using Microsoft PowerPoint 2016) of the experimental setup for cell adhesion assay. (**B**) Fluorescence photos of rat BMSCs attached to untreated (top) and plasma-treated (bottom) dense β-TCP disks before (left) and after (right) centrifugation. (**C**) The average numbers of cells attached to each dense β-TCP disk after initial adhesion but before centrifugation (left), those after centrifugation (center), and the adhesion rates (right) for untreated and plasma-treated dense β-TCP disks. The data for C are expressed as mean ± SD (n = 5). *p < 0.05, ** *p* < 0.01. Mann–Whitney U test. Cell morphologies of rat BMSCs attached to dense β-TCP disks were also analyzed after 3 h incubation for the initial adhesion and subsequent 24-h incubation with additional GM. (**D**) Fluorescence photos of the rat BMSCs after the incubation on untreated (left) and plasma-treated (right) dense β-TCP disks. (**E**) Comparison of cell areas (left), cell circularities, defined by Eq. (1) (center), and cell solidities, defined by Eq. (2) (right), between the untreated and plasma-treated disks. The cell area represents its size and the cell circularity and solidity characterize its shape. The data of E are expressed as mean ± SD with n = 1860 for “untreated” and n = 1440 for “plasma”. ****p < 0.0001. T-test.

Second, we investigated the effects of the plasma treatment on cell adhesion. Green fluorescent protein (GFP)-transgenic bone marrow stromal cells (GFP-BMSCs) were harvested from male GFP-transgenic rats. We seeded 5 × 10^3^ GFP-BMSCs on the surface of each dense β-TCP disk, incubated them for 30 min to initiate adhesion, and then detached weakly attached cells by centrifugation, as shown in Fig. 3A. Figure 3B shows fluorescence photos of rat BMSCs attached to dense β-TCP disks before and after centrifugation. As shown in Fig. 3C, both cell counts and adhesion rates were significantly higher for the plasma-treated disks than those of the untreated disks.

Additionally, we also analyzed morphologies of rat BMSCs attached to dense β-TCP disks. We seeded 5 × 10^3^ GFP-BMSCs on the surface of each dense β-TCP disk and incubated them for 3 h for initial adhesion. The cells were then further incubated with additional GM for 24 h. Figure 3D shows fluorescence images of the cells after the 24 h incubation. It is seen there that the morphology of attached cells on the plasma-treated β-TCP disk exhibits a significant increase in the area of cytoplasm (shown in green in the images). These images are used to evaluate the cell areas, cell circularities, defined in Eq. (1), and cell solidities, defined in Eq. (2), for the untreated and plasma-treated disks, which are shown in Fig. 3E. It should be noted that the sample numbers for the untreated and plasma-treated cases are n = 1884 and n = 1440, respectively, for the analysis of Fig. 3E, and the differences in the mean values are confirmed to be statistically significant with unpaired t-test analysis. As we visually observed in Fig. 3C,E shows that the average area of rat BMSCs is larger on the plasma-treated dense β-TCP disk than on the untreated disk (left), suggesting the enhancement of cell spreading on the plasma-treated surfaces. It also shows that the average circularity (center) and solidity (right) are lower on the plasma-treated dense β-TCP disk than on the untreated disk, indicating the presence of membrane protrusions of rat BMSCs on the plasma-treated dense β-TCP disk.

**Figure 4:**
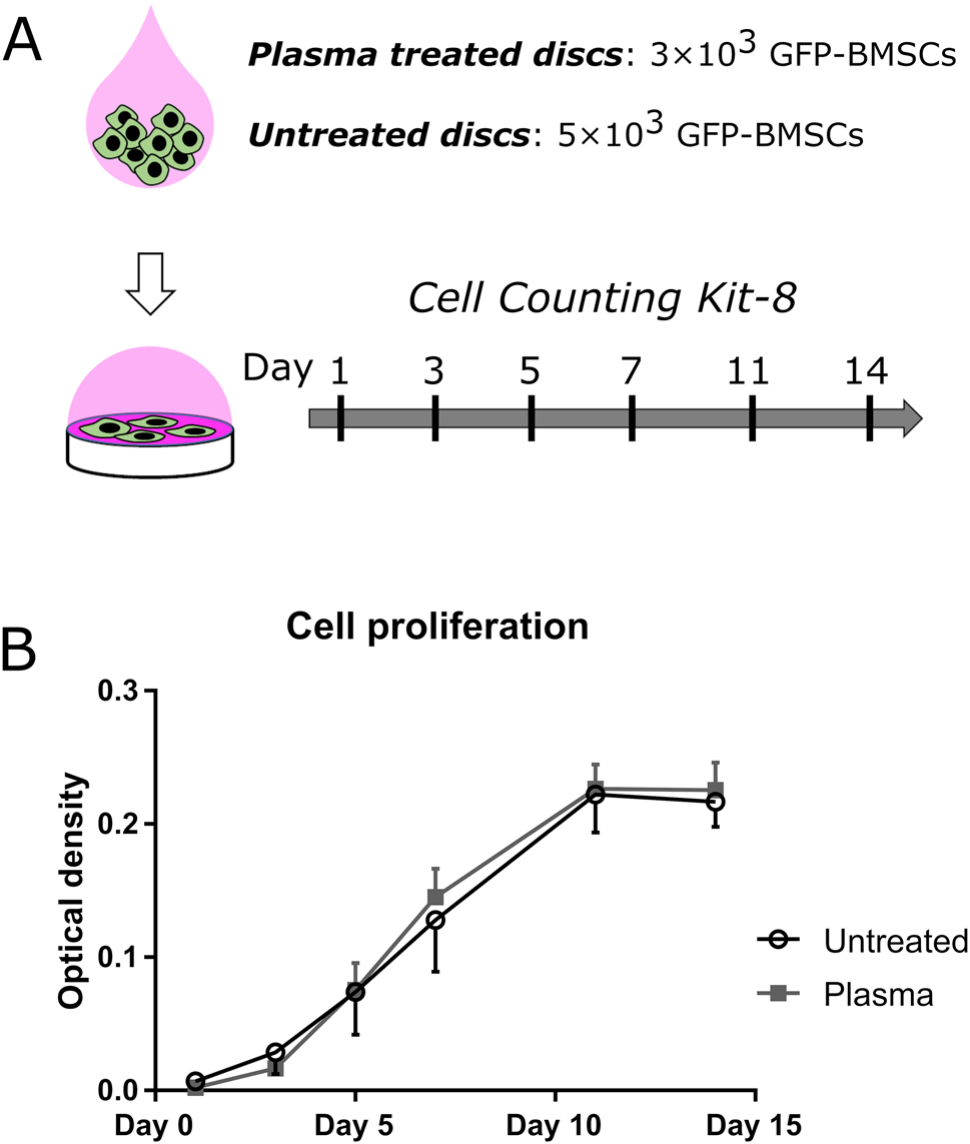
Proliferation assay for rat BMSCs on untreated and plasma-treated β-TCP disks. (**A**) Schematic diagram of the proliferation assay (drawn by using Microsoft PowerPoint 2016). (**B**) Cell proliferation curves for rat BMSCs on untreated (open circles) and plasma-treated (filled squares) β-TCP disks. All measurements were triplicate.

Third, we performed cell proliferation assays. Considering the difference in the initial cell adhesion, we made the initial number of cells the same by seeding different numbers of GFP-BMSCs on plasma-treated and untreated dense β-TCP disks and plotted the cell proliferation curves by serial Cell Counting Kit-8 (CCK-8) assays (Fig. 4A). It was found that the plasma treatment did not influence cell proliferation (Fig. 4B).

**Figure 5:**
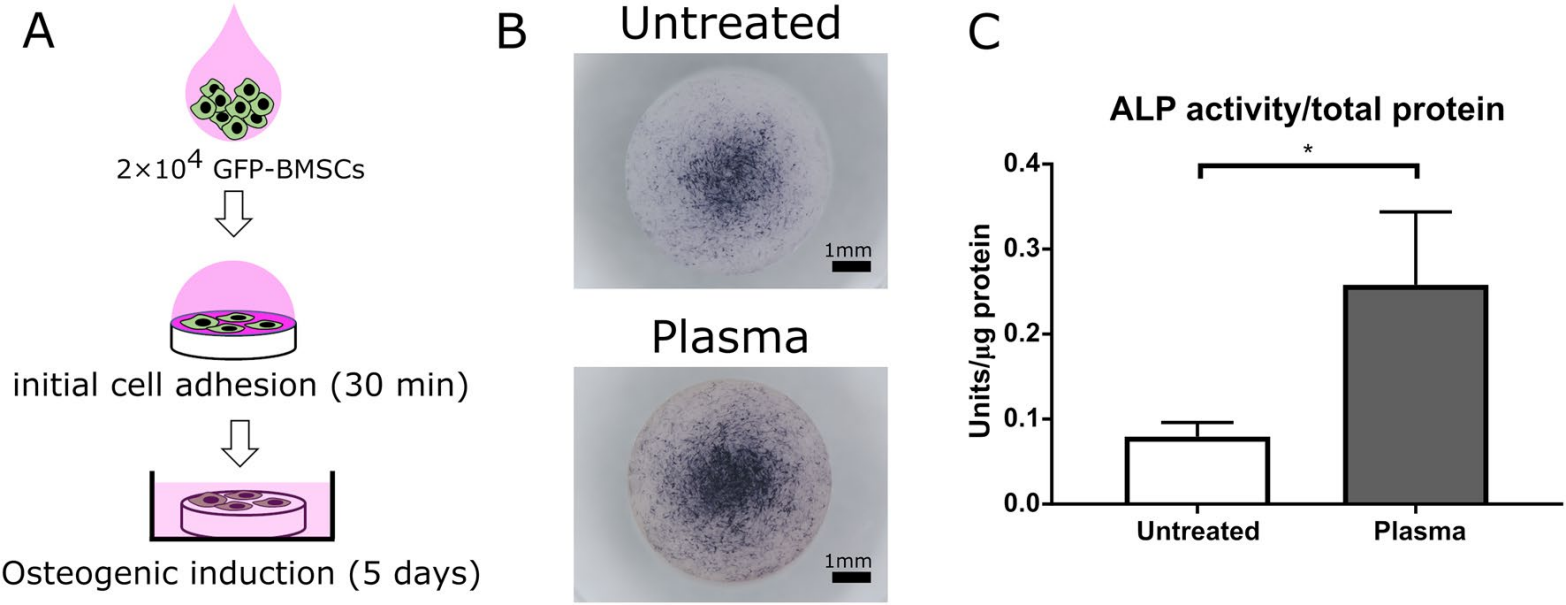
Osteogenic differentiation assay for rat BMSCs on untreated and plasma-treated β-TCP disks. (**A**) Schematic diagram of the osteogenic differentiation assay (drawn by using Microsoft PowerPoint 2016). (**B**) Macro photos of ALP-stained rat BMSCs sub-cultured on untreated (top) and plasma-treated (bottom) dense β-TCP disks. The black in the image represents the ALP stain. (**C**) The ALP activity values, as defined in the section Materials and methods, for untreated and plasma-treated β-TCP disks. The data here are expressed as mean ± SD (n = 3). *p < 0.05. T-test.

Last, to evaluate the overall osteogenesis including cell adhesion, proliferation, and osteogenic differentiation on plasma-treated and untreated dense disks, we seeded 2 × 10^4^ GFP-BMSCs on the surface of each dense disk and performed ALP assays after 5 days of osteogenic induction (Fig. 5A). The plasma treatment significantly improved osteogenesis as demonstrated by ALP staining (Fig. 5B) and ALP activity assay (Fig. 5C).

### Enhancement of in vivo new bone formation by the plasma treatment

**Figure 6:**
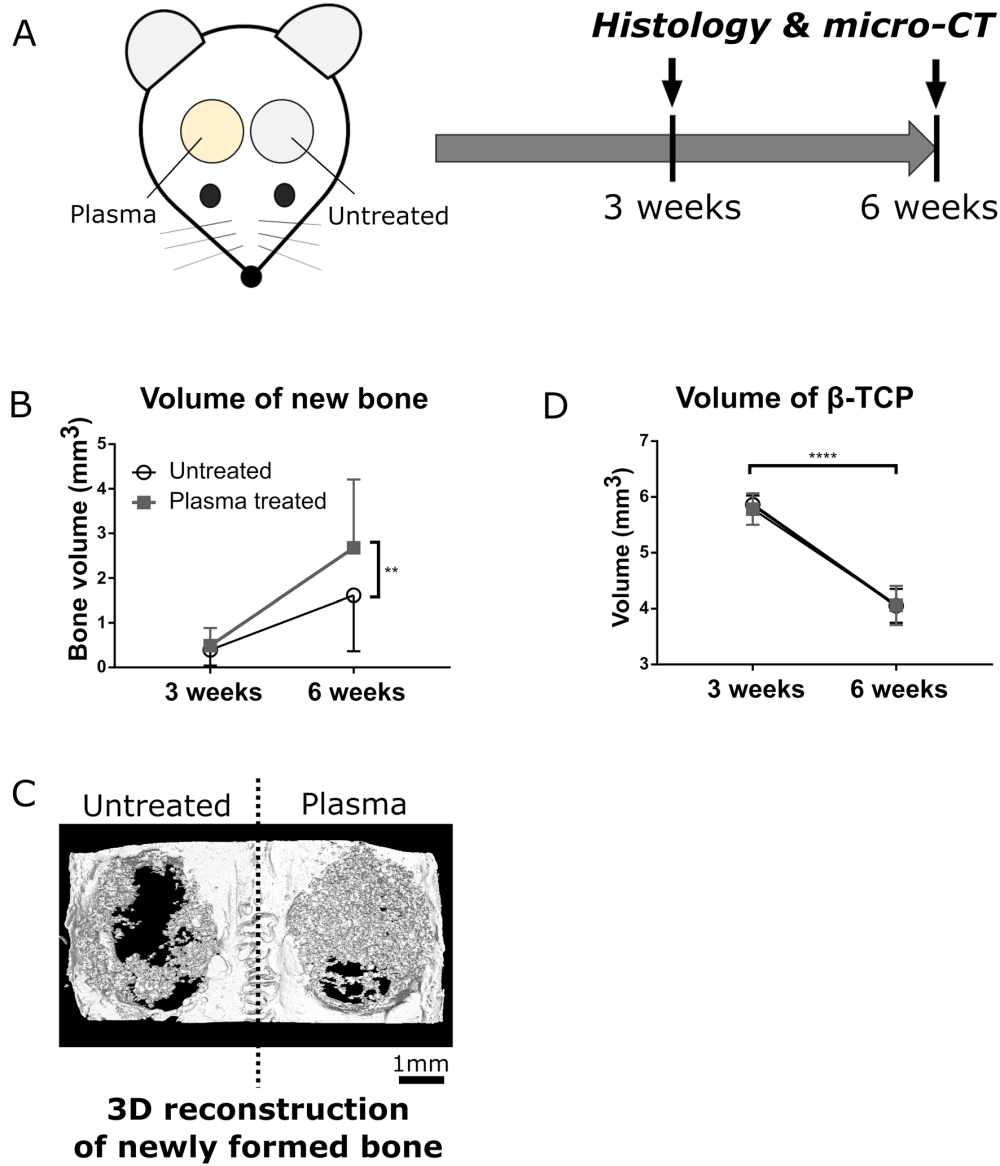
In vivo experiments on the new bone formation inside porous β-TCP disks. (**A**) Schematic diagram of the in vivo implantation of porous β-TCP disks and post-operative evaluations (drawn by using Microsoft PowerPoint 2016). (**B**) Micro-CT analysis of new bone volumes formed inside the untreated (open circles) and plasma-treated (filled squares) porous β-TCP disks at postoperative 3 and 6 weeks. (**C**) The 3D reconstruction of the calvarial bone implanted with untreated (left) and plasma-treated (right) porous β-TCP disks at postoperative 6 weeks. The gray grain-like structures indicate newly formed bone, and the white plate or slab-like structure with two circular holes represents the rat calvarial bone. The β-TCP disks are not depicted and therefore the volume occupied by β-TCP is represented by void space (black) in this image. (**D**) Micro-CT analysis of residual β-TCP volumes of the untreated (open circles) and plasma-treated (filled squares) porous β-TCP disks at postoperative 3 and 6 weeks. The data in B and D are expressed as mean ± SD (n = 5 for 3 weeks, n = 15 for 6 weeks). **p < 0.01, ****p < 0.0001. Wilcoxon matched-pairs signed-rank test.

Plasma-treated and untreated porous β-TCP disks were symmetrically transplanted into circular bone defects of rat calvarial bones. Histological evaluation and micro-CT analysis were performed at 3 and 6 weeks postoperatively (Fig. 6A).

As seen in Fig. 6B, at postoperative 3 weeks, the volume of new bone formed inside the plasma-treated porous β-TCP disk was evaluated and found not to differ essentially from that inside the untreated porous β-TCP disk. However, at postoperative 6 weeks, the volume of new bone formed inside the plasma-treated porous β-TCP disk was significantly higher than that inside the untreated porous β-TCP disk. The 3D-reconstructed image of new bone along with the host calvarial bone revealed abundant new bone formation in and around the plasma-treated porous β-TCP disk (Fig. 6C). In both untreated and plasma-treated groups, nearly one-third of the β-TCP was absorbed for the 3 weeks between the postoperative 3 and 6 weeks (Fig. 6D) with similar rates. Because the initial volumes of the β-TCP disks were essentially the same (about 9.8 mm^3^), the β-TCP absorption rate was unaffected by the plasma treatment.

**Figure 7:**
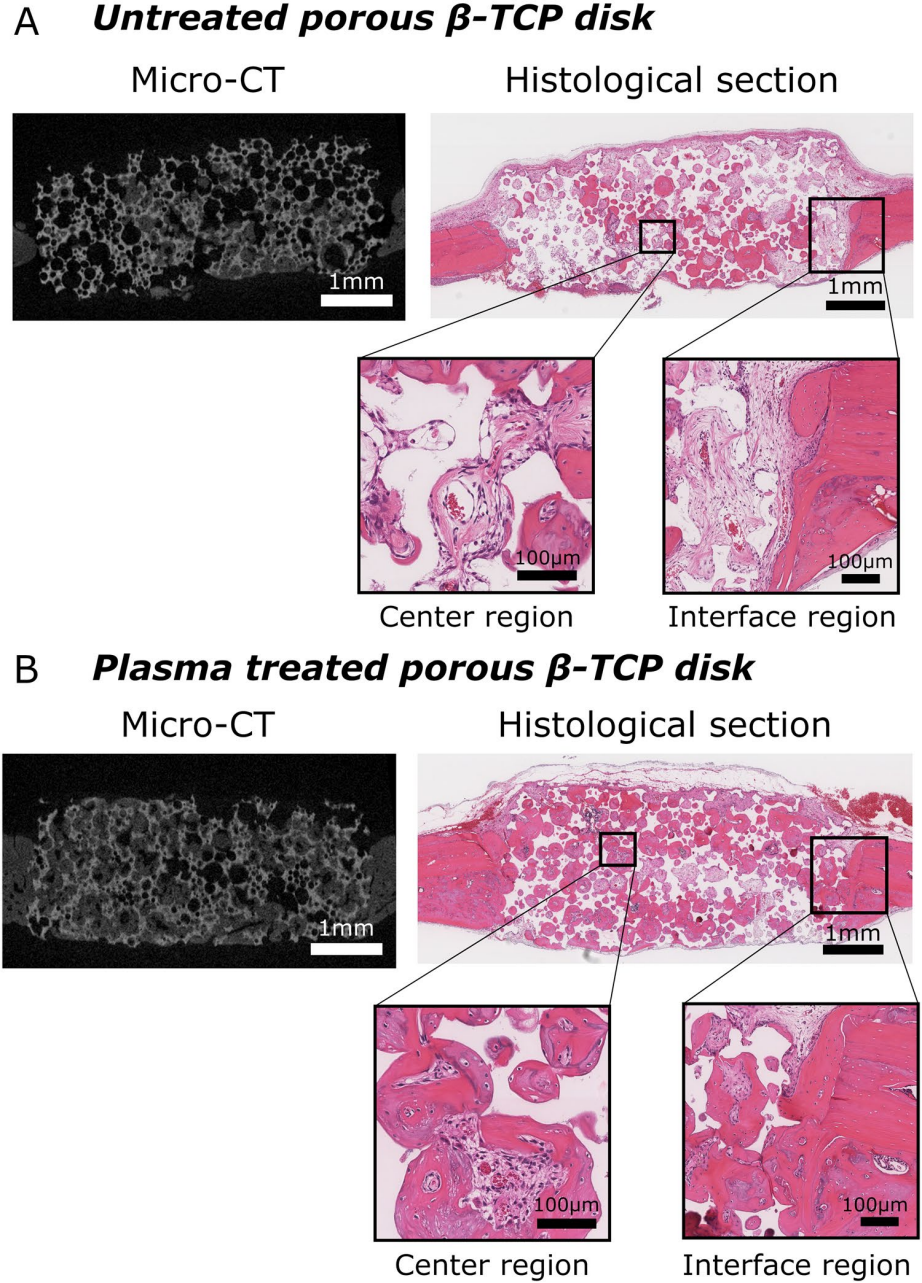
Histological sections at postoperative 6 weeks. (**A**) A cross-section image of an untreated porous β-TCP disk transplanted into a rat calvarial bone, reconstructed from its micro-CT scanning data (left). The white represents β-TCP, the gray represents the rat calvarial bone or newly formed bone, and the black represents soft tissues or the void of space. The corresponding histological section of the same porous β-TCP disk stained with H&E is shown on the upper right. The bright pink represents bony tissues, the pale pink represents soft tissues, the white represents the space of decalcified β-TCP or the void of space, and the nuclei of cells are stained in purple. Magnified images near the center and right interface region are also listed (bottom). (**B**) A cross-section image of a plasma-treated porous β-TCP disk transplanted into a rat calvarial bone, reconstructed from its micro-CT scanning data (left) and the corresponding histological section of the same porous β-TCP disk stained with H&E (upper right) and their magnified images (bottom). All images are at postoperative 6 weeks.

Figure 7 shows micro-CT slices and the corresponding histological sections of (A) untreated and (B) plasma-treated porous β-TCP disks transplanted into a rat calvarial bone at postoperative 6 weeks. In the micro-CT slices, the white represents β-TCP, the gray represents the rat calvarial bone or newly formed bone, and the black represents soft tissues or the void of space. In the histological sections, the bright pink represents bony tissues, the pale pink represents soft tissues, the white represents the space of decalcified β-TCP or the void of space, and the nuclei of cells are stained in purple. Magnified images near the center and right interface regions of the histological sections are also listed. More bone formation is observed in the plasma-treated porous β-TCP disk than in the untreated disk. The magnified histological images of the interface regions also indicate that the new bone formed in the plasma-treated β-TCP disk has achieved good interface union with the host bone whereas, at the interface between the untreated porous β-TCP disk and the host rat calvarial bone, the host bone is in contact only with soft tissues and has not achieved solid interface union with new bone.

## Discussion

In this study, we successfully formed amine-containing carbon polymers on β-TCP artificial bone surfaces by plasma polymerization using a gas mixture of CH_4_, N_2_, and He. This amine modification on β-TCP artificial bone surfaces enhanced in vitro cell adhesion and osteogenic differentiation as well as in vivo bone regeneration. The effects of amine-modified artificial bone surfaces on the tissue ingrowth and enhancement of osteogenic differentiation are considered to be the main mechanisms underlying this high bone regeneration capacity.

In the treatment of large bone defects with artificial bone, the successful introduction of cells and blood vessels at a substantial distance from the host bone remains challenging^26,27^. To treat “critical size” bone defects, pre-loading of bone or vascular-forming cells or vascular transplantation inside artificial bones has been attempted^28–31^. These methods usually require long and costly pre-treatment. In the present study, we instead chose a “chemical modification” approach and formulated amine-containing plasma polymerization on the outer and inner surfaces of porous artificial bone. The implantation of such plasma-treated artificial bone in a rat calvarial defect model demonstrated early tissue (cells and vessels) ingrowth. The enhanced cell infiltration by plasma treatment is considered to contribute to this early tissue ingrowth.

Amines, as functional groups, are hydrophilic. This is because, in an aqueous medium, amines become protonated and form positively charged functional groups –NH_*x*_^+^ (*x* = 1–3, depending on how many C atoms the center N atom is bonded with), which attract highly polarized water molecules. The present study revealed that amine modification of porous β-TCP surfaces enhanced the infiltration of cell suspension dropped on them (Supplementary Fig. 1). Notably, the plasma-treated β-TCP absorbed tissue fluid and rapidly became wet, in contrast to non-treated β-TCP, which remained almost completely dry during the implantation (Supplementary Fig. 2). Although the volumes of new bone formed at postoperative 3 weeks were observed to be similar between the plasma-treated and untreated porous β-TCP disks (Fig. 6B), the histological sections at postoperative 3 weeks revealed abundant infiltrated cells and tissue formation in the plasma-treated disks (Supplementary Fig. 3).

We demonstrated that β-TCP coated with amine-containing plasma-polymerized films enhanced in vitro osteoblastic differentiation and increased the volume of new bone inside the interconnected pores of artificial bones in vivo. Several effects provided by the amine modification must have contributed to this enhanced bone formation.

Amine modification of a surface is known to strengthen cell adhesion by enhancing integrin binding, which is required for osteoblastic differentiation^32–34^. In detail, cell adhesion is mainly mediated by the binding of cellular integrins and adhesive proteins such as fibronectin in the extra-cellular matrix. The positive charges of amines can increase the density of fibronectin and change its conformation^35^. These changes in fibronectin strengthen the cell adhesion by increasing the binding to integrins^36,37^ and trigger rapid phosphorylation of focal adhesion-associated tyrosine kinase (FAK)^38^, subsequently triggering extracellular signal-regulated kinase (ERK)/mitogen-activated protein kinase (MAPK) signaling to upregulate Runt-related transcription factor 2 (Runx2), which is a master regulator of osteoblastic differentiation^39–45^.

The type of change in cell morphology that we observed on the plasma-treated β-TCP, i.e., the increase of cell areas and the decrease of circularity and solidity, is known to facilitate the osteogenic differentiation of rat BMSCs^46^. This is similar to the earlier observation that human mesenchymal stem cells exhibiting a spreading, rather than maintaining round shapes, appear inclined toward an osteogenic lineage^47^. This is considered to be caused by the upregulation of Ras homolog family member A (RHOA), a transcription factor that regulates the actin cytoskeleton and increases osteogenesis^46–48^.

The basicity of amines can also improve osteogenesis by increasing the interfacial pH^49^. A high pH environment around implant materials has been reported to enhance osteoblastic differentiation^49,50^.

Our study also showed that the plasma treatment enhanced the osteogenic differentiation without affecting cellular proliferation (Fig. 2B). In general, cellular proliferation and osteogenic differentiation do not necessarily occur simultaneously. Indeed, earlier studies also demonstrated the enhancement of osteogenic differentiation without enhanced cellular proliferation for different types of cells under different conditions^6,51,52^.

The enhanced cell adhesion, change in cell morphology, and possibly the increase of interfacial pH due to the presence of amine groups on β-TCP artificial bone surfaces are thus consistent with, and underlie, the high bone regeneration capacity achieved by the plasma polymerization on the artificial bones.

The major limitation of this study is, however, that we investigated the bone regeneration capacity only with a rat calvarial defect model. The calvaria has a rich blood supply and therefore offers a supportive environment for bone regeneration. A further investigation on bone regeneration under more stringent conditions, e.g., that for malunion of fractures, is desirable. Furthermore, as the size of artificial β-TCP bones designed for the rat calvarial defect model was small, we were only able to evaluate the bone volume with a high-resolution micro-CT, but unable to obtain other important bone parameters such as trabecular numbers and thicknesses or to perform biomechanical tests to evaluate mechanical strengths of the regenerated bone.

Despite such limitations, the findings of this study indicated that the rapid cell infiltration into the interconnected pores of artificial bones and the enhanced osteoblastic differentiation capability due to the presence of plasma-polymerized films on the artificial bone surfaces contributed to the early tissue ingrowth and high bone regeneration inside and around the plasma-treated artificial bones. Considering the fact that no commercial artificial bone is currently available that possesses a satisfactory osteogenic capacity, we expect these characteristics of amine-modified artificial bones to pave the way to the development of a safe and more effective treatment of large bone defects.

## Supplementary Information

Supplementary Information 1.

Supplementary Video 1.

## Data Availability

The datasets generated and/or analyzed during the current study are available from the corresponding authors on reasonable request.
