# Supplementary material for: Amine modification of calcium phosphate by low-pressure plasma for bone regeneration
Source: Sci Rep. 2021 Sep 9;11:17870. doi: 10.1038/s41598-021-97460-8 (PMC8429709; doi:10.1038/s41598-021-97460-8)

Supplementary materials to

Amine modification of calcium phosphate by low-pressure plasma for bone regeneration

Joe Kodama[1,‡], Anjar Anggraini Harumningtyas[2,3,‡], Tomoko Ito[2], Miroslav Michlíček[4], Satoshi Sugimoto[2], Hidekazu Kita[2], Ryota Chijimatsu[1,5], Yuichiro Ukon[1], Junichi Kushioka[1], Rintaro Okada[1], Takashi Kamatani[1], Kunihiko Hashimoto[1], Daisuke Tateiwa[1], Hiroyuki Tsukazaki[1], Shinichi Nakagawa[1], Shota Takenaka[1], Takahiro Makino[1], Yusuke Sakai[1], David Nečas[6], Lenka Zajíčková[6,7], Satoshi Hamaguchi[2,*], Takashi Kaito[1,*]

[1] Department of Orthopaedic Surgery, Osaka University Graduate School of Medicine, Osaka, 565-0871, Japan

[2] Center for Atomic and Molecular Technologies, Graduate School of Engineering, Osaka University, 2-1 Yamadaoka, Suita, Osaka 565-0871 Japan

[3] Center for Accelerator Science and Technology, National Nuclear Energy Agency of Indonesia (BATAN), Jalan Babarsari Kotak Pos 6101 ykbb Yogyakarta 55281, Indonesia

[4] Department of Physical Electronics, Faculty of Science, Masaryk University, Kotlarska 2, Brno 61137, Czech Republic

[5] Bone and Cartilage Regenerative Medicine, Graduate School of Medicine, The University of Tokyo, 7-3-1 Hongo, Bunkyo-ku, Tokyo, 113-8655, Japan

[6] CEITEC – Central European Institute of Technology, Brno University of Technology, Purkynova 123, Brno 61200, Czech Republic

[7] Department of Condensed Matter Physics, Faculty of Science, Masaryk University, Kotlarska 2, Brno 61137, Czech Republic

[‡] These authors contributed equally to this work.

[*]Corresponding authors

Takashi Kaito, MD, PhD.

Department of Orthopaedic Surgery, Osaka University Graduate School of Medicine, 2-2 Yamadaoka, Suita, Osaka 565-0871, Japan.

Tel: +81-6-6879-3552

Fax: +81-6-6879-3559

E-mail: takashikaito@ort.med.osaka-u.ac.jp

Satoshi Hamaguchi, PhD.

Center for Atomic and Molecular Technologies (CAMT),
Graduate School of Engineering, Osaka University,
2-1 Yamadaoka, Suita, Osaka 565-0871, Japan.
TEL: +81-6-6879-7913 (06-6879-7913)
FAX: +81-6-6879-7916 (06-6879-7916)
E-mail: hamaguch@ppl.eng.osaka-u.ac.jp

Supplementary Movie 1 is provided as a separate file, which recorded the infiltration of cell suspensions into the porous β-TCP disks.

Supplementary Figure 1. Plasma treatment enhanced the hydrophilicity of porous β-TCP disks, resulting in faster infiltration of cell suspension and the cells therein. (A) Snapshots of cell suspensions dropped on untreated (left) and plasma-treated (right) porous infiltration β-TCP disks. From top to bottom; 1 sec. before the cell suspensions were dropped (t = -1 sec); the moment they were dropped on the surfaces (t = 0 sec); states of cell suspension droplets 2 sec. after they were dropped (t = 2 sec). It is seen that the plasma-treated porous β-TCP disk completely absorbed the cell suspension droplet. (B) Cross sections of untreated (left) and plasma-treated (right) porous β-TCP disks after cell suspensions were dropped on them. Crystal violet staining shows the cells infiltrated and attached to the inner pore surfaces. It is seen that more cells are infiltrated more deeply into the plasma-treated porous β-TCP disk. The plasma-treated β-TCP disk was plasma treated on both sides for 30 min each.

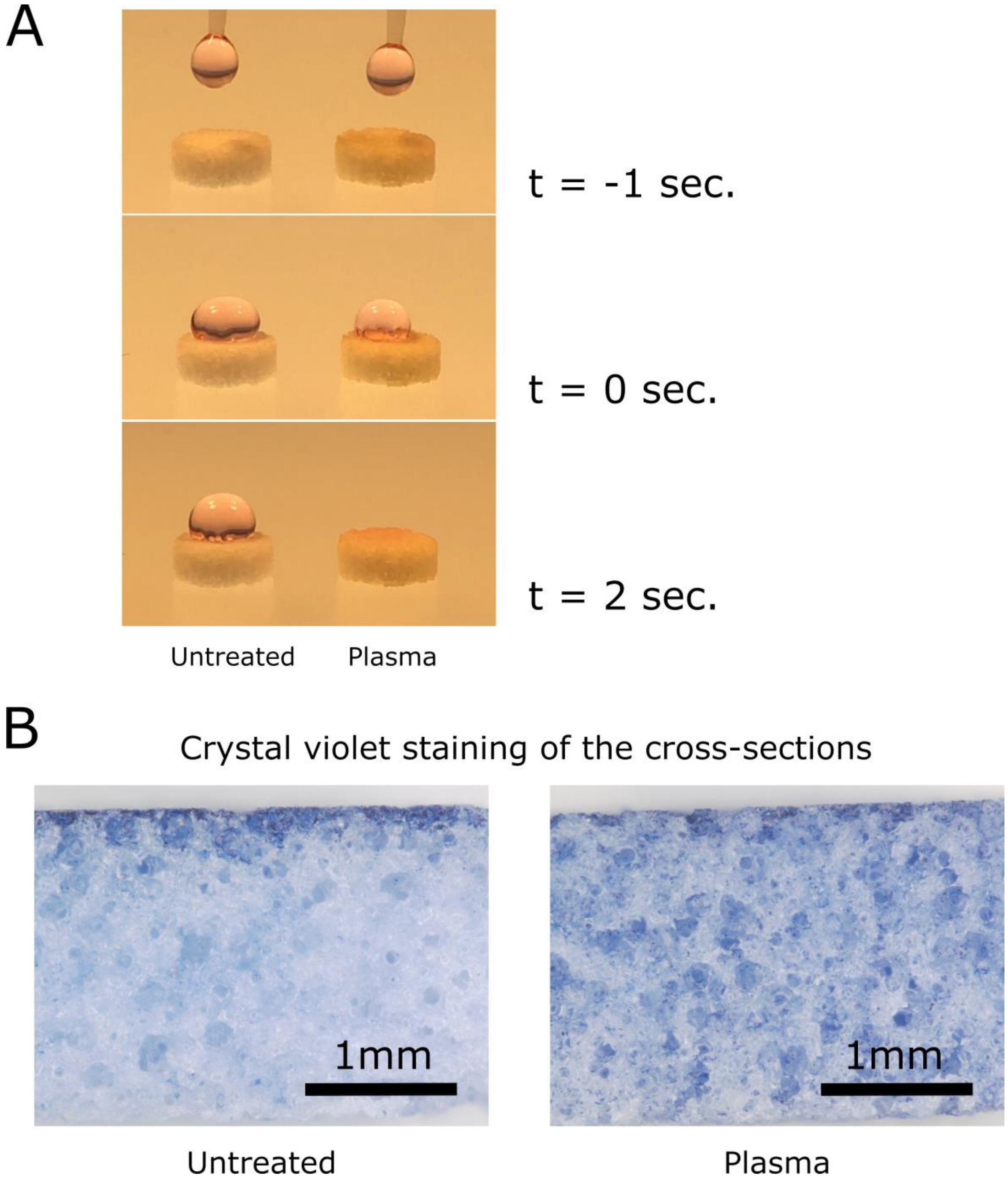
A
t = -1 sec.
t = 0 sec.
t = 2 sec.
Untreated
Plasma
B
Crystal violet staining of the cross-sections
1mm
1mm
Untreated
Plasma

Supplementary Figure 2. Photo of the implantation of plasma-treated (left) and untreated (right) porous β-TCP disks into rat calvarial bone defects. The plasma-treated disk shows faster blood infiltration.

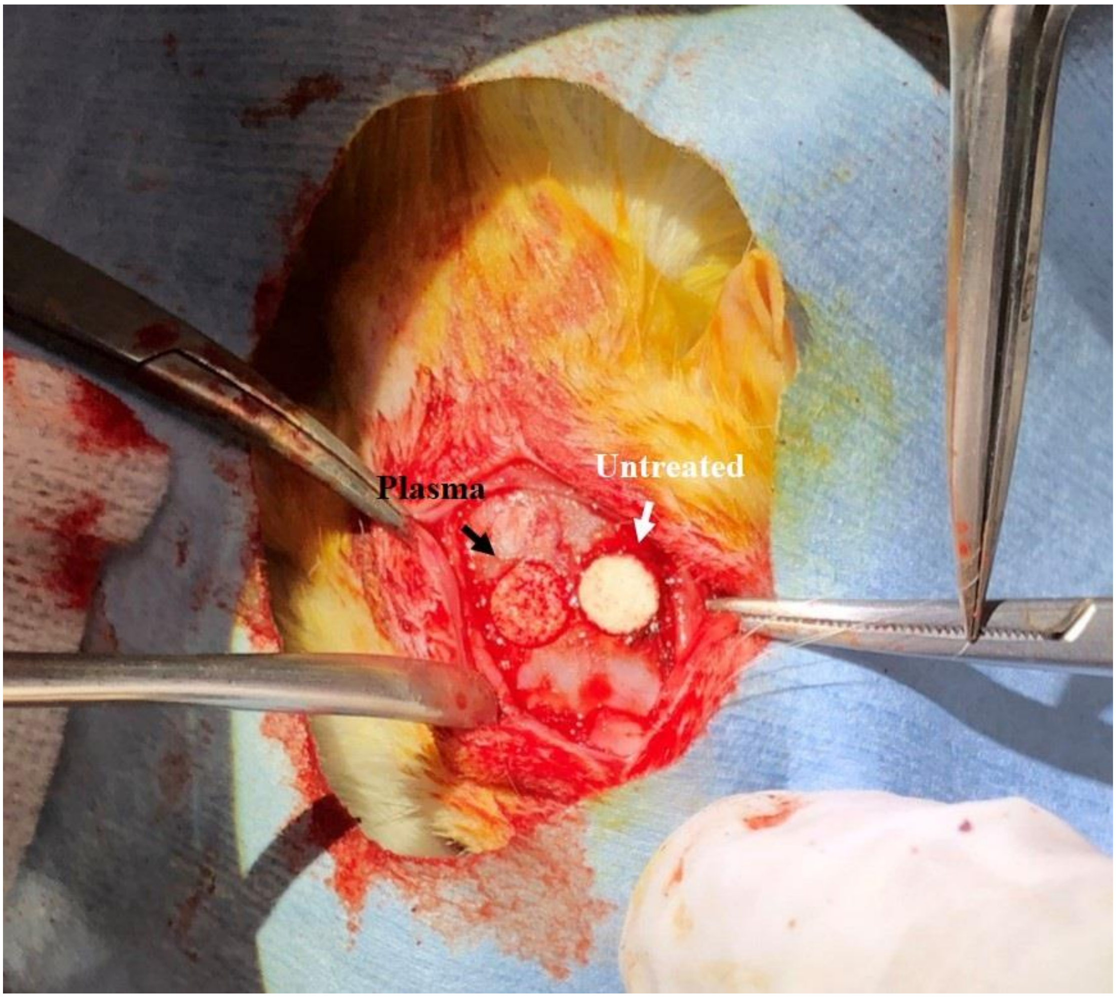

Supplementary Figure 3. Histological sections of untreated (top) and plasma-treated (bottom) porous β-TCP disks implanted into rat calvarial bone defects; at postoperative 3 weeks. The plasma-treated disk shows denser H&E staining in the inner-pores than the untreated disk, indicating faster new bone formation in the plasma-treated disk.

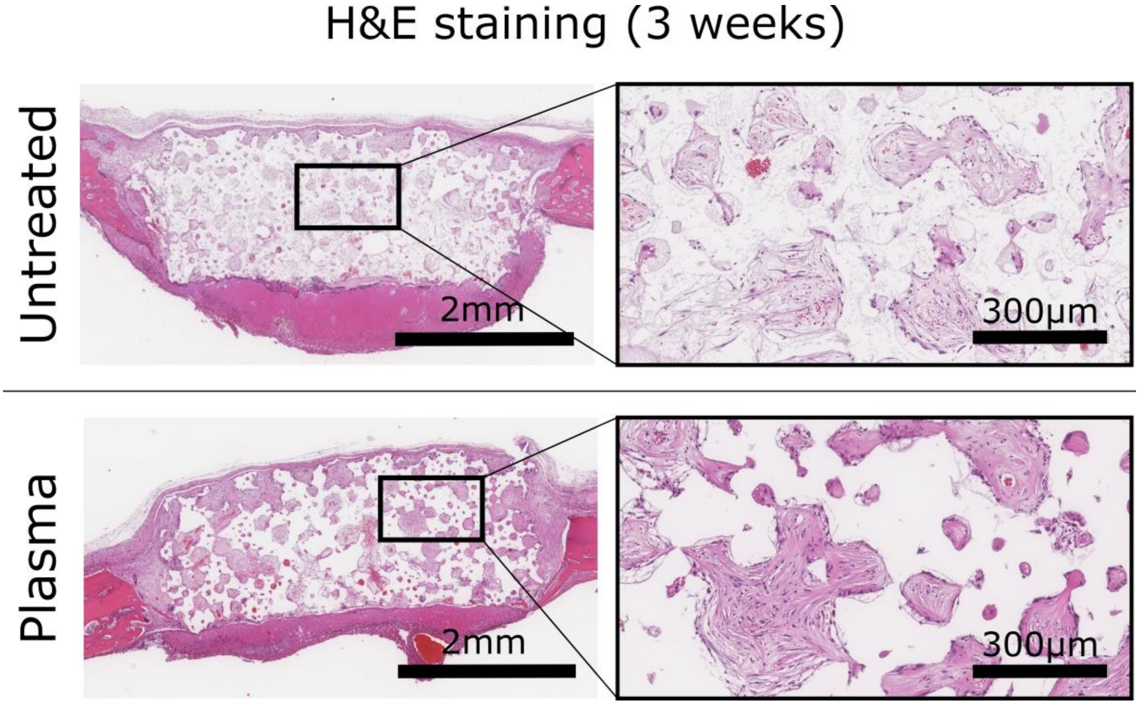

Supplement: Supplementary file 1 — Supplementary Information 1. [file 41598_2021_97460_MOESM1_ESM.pdf]
